# Regulatory T Cells in Invasive Breast Cancer: Prognosis, Mechanisms and Therapy

**DOI:** 10.3390/cancers17193172

**Published:** 2025-09-29

**Authors:** Aizhang Xu, Sama Ayoub, Haijun Zhang, Yuhang Wu, Marcellino Rau, Xiaojing Ma

**Affiliations:** 1 Department of Microbiology and Immunology, Weill Cornell Medicine, New York, NY 10021, USA; aix4001@med.cornell.edu (A.X.); haz2004@med.cornell.edu (H.Z.); 2 Weill Cornell Medicine-Qatar, Doha 24144, Qatar; sna4001@qatar-med.cornell.edu; 3 Middlebury College, Middlebury, VT 05753, USA; yuhangw@middlebury.edu; 4 John Jay College of Criminal Justice, New York, NY 10019, USA; marcellino.rau@jjay.cuny.edu; 5 Sandra and Edward Meyer Cancer Center, Weill Cornell Medicine, New York, NY 10021, USA

**Keywords:** Regulatory T cells (Tregs), invasive breast cancer, tumor microenvironment (TME), immunosuppression, cancer immunology, FoxP3, tumor-infiltrating lymphocytes (TILs), immunotherapy, prognosis, triple-negative breast cancer (TNBC), HER2-positive breast cancer, hormone receptor-positive breast cancer, immune evasion, checkpoint inhibitors, metastasis, Artificial Intelligence

## Abstract

**Simple Summary:**

Breast cancer is one of the most common and deadly cancers in women, and its treatment remains challenging because tumors can hide from the body’s natural defenses. A special group of immune cells called regulatory T cells normally prevent harmful inflammation, but in breast cancer they are often taken over by the tumor to block protective immune responses. This review explains what is currently known about how these cells influence breast cancer growth, spread, and resistance to therapy. We also describe new approaches being studied to control or reprogram regulatory T cells so that the immune system can better fight cancer. By bringing together findings from laboratory studies and clinical research, our goal is to highlight opportunities for more precise treatments. Understanding the role of regulatory T cells may help doctors develop strategies that improve survival and quality of life for patients.

**Abstract:**

Regulatory T cells (Tregs) are a specialized subset of CD4+ T lymphocytes essential for maintaining immune tolerance and preventing autoimmunity. However, in breast cancer, tumors exploit Tregs to establish an immunosuppressive microenvironment that enables immune evasion, accelerates progression, and contributes to therapeutic resistance. This review synthesizes current evidence on the role of Tregs in invasive breast cancer (IBC), highlighting their prognostic significance across molecular subtypes, mechanisms of immune suppression, and impact on treatment response. We integrated mechanistic and clinical insights to discuss opportunities for Treg-targeted therapeutic strategies, with attention paid to challenges such as autoimmunity, compensatory resistance, and subtype-specific heterogeneity. Finally, we outline future directions, including biomarker-driven precision medicine, novel therapeutic combinations, advanced preclinical models, as well as potential artificial intelligence-assisted approaches that aim to selectively disrupt tumor-promoting Treg functions while preserving the systemic immune balance.

## 1. Introduction

### 1.1. Clinical and Molecular Heterogeneity of Invasive Breast Cancer

Invasive breast cancer (IBC) encompasses a spectrum of diseases with distinct histological and molecular features. The most common histological subtype, invasive ductal carcinoma (IDC), accounts for approximately 70–80% of cases, followed by invasive lobular carcinoma (ILC) (~10–15%), with other rare subtypes comprising the remainder [1]. Besides histology, molecular classification based on estrogen receptor (ER), progesterone receptor (PR), and HER2 expression defines clinically relevant subtypes: luminal A, luminal B, HER2-enriched, and triple-negative breast cancer (TNBC) [2]. These subtypes differ in aggressiveness, therapeutic responsiveness, and prognosis. Luminal A cancers are generally low-grade and endocrine-responsive, whereas luminal B cancers are more aggressive, requiring chemotherapy in addition to endocrine therapy [3]. HER2-enriched tumors, once associated with poor outcomes, now benefit from effective HER2-targeted therapies [4]. TNBC remains the most challenging subtype, with high aggressiveness, limited targeted therapies, and poor prognosis despite chemotherapy and emerging immunotherapy options [5]. Intra-tumor heterogeneity further complicates treatment, underscoring the need for precision medicine approaches [6].

### 1.2. The Breast Tumor Immune Microenvironment

The tumor immune microenvironment (TME) shapes disease trajectory and treatment outcomes [7]. Tumor-infiltrating lymphocytes (TILs), particularly CD8+ cytotoxic T cells, are associated with favorable prognosis and immunotherapy responsiveness [8,9]. Conversely, immunosuppressive populations, including Tregs, myeloid-derived suppressor cells (MDSCs), and tumor-associated macrophages (TAMs), drive immune evasion and therapeutic resistance [10]. TNBC is typically more immunogenic, with higher TIL density and better responses to checkpoint blockade, whereas luminal tumors remain relatively immunologically “cold.” HER2-positive tumors occupy an intermediate position but respond to combined HER2-targeted and immune-based therapies [11,12]. Mechanisms of immune evasion include checkpoint molecule upregulation [Programmed Death Ligand 1 (PD-L1), Cytotoxic T Lymphocyte Antigen-4 (CTLA-4)], immunosuppressive cytokine secretion [Interleukin-10 (IL-10), Transforming Growth Factor B (TGF-β)], and impaired antigen presentation [13]. Advances in single-cell sequencing and spatial transcriptomics have provided unprecedented resolution of TME complexity, revealing heterogeneity in immune composition and function even within individual tumors [14,15].

### 1.3. Regulatory T Cells: Guardians of Tolerance, Enablers of Evasion

Tregs, defined by CD4+CD25+FOXP3+ expression, play a dual role. Physiologically, they maintain immune homeostasis and prevent autoimmunity [16]. In cancer, they suppress effector immune responses through multiple mechanisms: secretion of IL-10, TGF-β, and IL-35 [17]. checkpoint engagement [(CTLA-4, Programmed Death 1 (PD-1), Lymphocyte-Activation Gene 3 (LAG-3), T cell Immunoglobulin and Mucin Domain-containing Protein 3 (TIM-3)] [18,19]. IL-2 consumption [20]. metabolic disruption (CD39/CD73-mediated adenosine generation, Indoleamine 2,3-dioxygenase (IDO)-mediated tryptophan depletion) [21,22]. and direct cytolysis of effector cells [23]. Tumor-associated Tregs adapt to hypoxia and metabolic stress, reinforcing suppression [24]. Their high prevalence in breast tumors correlates with aggressive disease, poor survival, and therapeutic resistance [25]. However, paradoxical associations exist in specific subtypes, reflecting context-dependent functions [26].

### 1.4. Rationale for Targeting Tregs

High Treg density in breast cancer is associated with advanced stage, lymph node involvement, and poor outcomes [25]. The selective depletion or functional reprogramming of Tregs enhances effector T cell activity in preclinical models and can synergize with immunotherapy [27,28]. Targeting Tregs in breast cancer is rationalized by the prospect of reinvigorating anti-tumor immunity. The immunosuppressive influence of Tregs can blunt the efficacy of immunotherapies (such as checkpoint inhibitors or cancer vaccines); hence, reducing Treg-mediated suppression could enhance the body’s natural immune response or improve responses to treatments [29]. Clinical strategies include low-dose chemotherapy (cyclophosphamide), anti-CD25 or anti-CCR4 (C-C motif chemokine receptor 4) antibodies, and checkpoint inhibitors [30,31]. The challenge lies in balancing anti-tumor efficacy with the preservation of immune tolerance, as systemic Treg depletion risks autoimmunity [32]. Tumor-specific Treg markers [(CCR8, T-cell immunoreceptor with Ig and ITIM domains (TIGIT)] may offer more selective therapeutic avenues [33,34,35,36].

## 2. Tregs in Breast Cancer Biology

### 2.1. Recruitment and Accumulation of Tregs in the Tumor Microenvironment

Tregs are recruited to breast tumors through chemokine signaling and tumor-derived factors. Chemokines (also known as chemotactic cytokines) are a large family of small proteins that signal through cell surface chemokine receptors and are best known for their ability to stimulate the migration of cells, including Treg cells in the tumor microenvironment. More specifically, cancer cells release chemokines to attract Treg cells to tumor sites, further facilitating cancer growth [37]. While tumor-derived factors encompass cytokines, growth factors, metabolites, and glycoproteins, they are all responsible for establishing immunosuppressive networks that result in tumor growth [38].

(a) *CCL22-CCR4 axis.* The CCL22-CCR4 axis is a key pathway, with tumor cells and stromal cells secreting the chemokine CCL22 to attract CCR4+ Tregs. Ovarian tumor Treg cells express the functional CCR4 receptor, which binds to CCL22 and migrates toward the tumor microenvironmental CCL22 in vitro and in vivo [39].

(b) *CXCL12-CXCR4 axis.* Hypoxia, a condition of low oxygen levels and a hallmark of the TME, further enhances the recruitment of Tregs through the CXCL12–CXCR4 axis. Under hypoxic stress, CXCR4 expression on Tregs is upregulated via the activation of the hypoxia-inducible factor (HIF) pathway. Tumor cells secrete the chemokine CXCL12, which attracts Tregs by binding to its receptor, CXCR4. This not only increases Treg migration but also boosts their immunosuppressive function by promoting the expression of the transcriptional regulator FOXP2. Notably, this pathway may be especially active in basal-like breast cancers, which are characterized by a heightened hypoxic response [40].

(c) *Prostaglandin E2.* Prostaglandin E2 (PGE2), a member of the prostanoid family of lipids, is produced by cancerous stromal cells and plays a role in promoting tumor cell proliferation. Through the COX-2/PGE2 signaling pathway, in which COX enzymes convert arachidonic acid into prostaglandins, PGE2 stimulates FOXP3 expression, thereby enhancing the immunosuppressive properties of Tregs. In vivo studies have demonstrated that inhibiting COX-2 results in a reduction in Treg frequency and activity, as well as diminished FOXP3 expression in tumor-infiltrating lymphocytes (TILs), leading to a corresponding decrease in tumor weight. However, these effects can be reversed by either the adoptive transfer of Treg cells or the administration of PGE2 to mice treated with COX-2 inhibitors. This highlights the crucial role of PGE2 in driving FOXP3 expression and enhancing Treg function through the COX-2/PGE2 signaling pathway [41].

### 2.2. Mechanisms of Suppression

Once recruited into the tumor microenvironment (TME), Tregs deploy a wide repertoire of overlapping suppressive pathways that collectively blunt anti-tumor immunity. These mechanisms include cytokine secretion, checkpoint receptor engagement, metabolic competition, and direct cytotoxicity. Each contributes to a tolerogenic ecosystem that enables breast tumors to persist and evade immune surveillance.

(a) *Cytokine-mediated suppression.* The TME harbors sophisticated regulatory T cell populations that employ multi-pathway cytokine-mediated suppression networks to systematically dismantle anti-tumor immunity. Tregs secrete immunosuppressive cytokines, including interleukin-10 (IL-10), transforming growth factor-β (TGF-β), and often IL-35. These cytokines blunt anti-tumor immunity by inhibiting dendritic cell (DC) maturation and function, suppressing CD4+ helper T cell activity, and blocking the generation of tumor-specific CD8+ cytotoxic T lymphocytes [42].

Sawant et al. [42]. used single-cell analysis to demonstrate that IL-10^+^ and IL-35^+^ Treg subpopulations cooperatively promote tumor T cell exhaustion through distinct but complementary molecular programs, with IL-10 and IL-35 synergistically driving T cell exhaustion by upregulating multiple inhibitory receptors on CD8+ tumor-infiltrating lymphocytes. The discovery that these cytokines operate through convergent but mechanistically distinct pathways represents a significant advance in the understanding of Treg biology. Both IL-10 and IL-35 upregulate BLIMP1 expression in target T cells, but IL-10 primarily affects effector cell differentiation, while IL-35 disrupts memory formation [42]. This compartmentalization allows tumor infiltrating Tregs to simultaneously prevent both immediate anti-tumor responses and long-term immunological memory, explaining the persistent immunosuppression observed in established tumors.

TGF-β signaling through Smad2/3 pathways fundamentally reprograms dendritic cell function, suppressing the expression of MHC class II molecules, CD80, CD86, and CD83, while inhibiting IL-12 and TNF-α production [43,44,45]. Experimental neutralization studies have demonstrated that anti-TGF-β antibodies can restore cytokine production to control levels. Latent TGF-β is activated by integrin β8. Anti-integrin β8 treatment enhances CD8+ T cell cytotoxic functions by 2-5-fold in patient ex vivo studies. This pathway creates tolerogenic rather than immunostimulatory dendritic cells, preventing effective T-cell priming and perpetuating immunosuppression. TGF-β is induced by IL-33 stimulation. The IL-33/ST2 pathway has emerged as another critical mechanism, with ST2-expressing Tregs showing enhanced suppressive functions by inducing IL-10 and TGF-β1 [46]. Additionally, membrane-bound TGF-β on Tregs can directly inhibit nearby CD8+ T cells and DCs, thereby further dampening immune responses [44]. The net result is a localized tolerance in the TME that allows cancer cells to escape immune surveillance. Breast tumors with elevated TGF-β activity tend to exhibit stronger regulatory T cell signatures coupled with immune-excluded phenotypes, including poor CD8+ infiltration and reduced effector function [47,48].

In IBC, IL-10 limits dendritic-cell (DC) maturation/IL-12 production through STAT3 signaling, curtailing type-1 priming in TME [49]. IL-35 induces a dysfunctional/exhausted state in CD8+ T cells and can propagate “infectious tolerance” [50,51]. TGF-β engages Smad2/3 to inhibit Teff differentiation and promotes tissue-resident Treg programs; TGF-β-rich breast tumors show stronger Treg signatures and immune exclusion [48]., providing a rationale for combinatorial TGF-β blockade.

In summary, cytokine secretion enables Tregs to suppress anti-tumor immunity at multiple levels, including antigen presentation, effector activity, and memory formation, ensuring persistent immunosuppression.

(b) *Immune checkpoint engagement.* Tregs constitutively express elevated levels of inhibitory receptors, which allows them to engage in immune checkpoints and suppress the activation of effector cells. One critical molecule is Cytotoxic T-Lymphocyte Antigen-4 (CTLA-4), which Tregs use to outcompete conventional T cells for binding to the co-stimulatory ligands CD80/86 on antigen-presenting cells. By sequestering CD80/86, Tregs downregulate the co-stimulatory “signal 2” required for the activation of naive T cells [44]. Recent mechanistic insights from Tekguc et al. [52]. revealed that Treg-expressed CTLA-4 depletes CD80/CD86 through trogocytosis, releasing free PD-L1 on antigen-presenting cells, providing a novel mechanistic link between CTLA-4 and PD-L1 pathways that explains the synergistic effects of combination checkpoint inhibition. This leads to insufficient activation of conventional T-cells, contributing to T-cell anergy or deletion. Moreover, Treg-expressed CTLA-4 actively reprograms nearby DCs: CTLA-4 engagement induces DCs to upregulate IDO [53]. Depletion of tryptophan and accumulation of kynurenine create an inhospitable environment for effector T cells (which require tryptophan for proliferation), thereby reinforcing immune suppression [53]. Tregs also express other checkpoint molecules such as LAG-3 and TIM-3; LAG-3 binding to MHC class II on DCs can directly impair antigen presentation and drive DCs toward tolerance (further amplifying suppression via IDO-dependent mechanisms) [44]. In parallel, TIM-3 marks a highly suppressive Treg subset, TIM-3⁺ Tregs exhibit enhanced FOXP3, LAG-3, PD-1, and CTLA-4 expression and more potently inhibit T helper responses in vitro [54]. and TIM-3⁺ tumor-infiltrating Tregs significantly suppress autologous CD8+ T-cell proliferation in human tumors [55]. Additionally, tumor-infiltrating Tregs often upregulate Programmed Death-1 (PD-1) on their surface. PD-1 on Tregs can interact with PD-L1 (abundantly expressed by tumor cells and myeloid cells in the tumor milieu), which appears to sustain Treg suppressive activity and deliver an inhibitory signal that dampens surrounding T cell responses [56]. Notably, the role of PD-1 signaling in Tregs is complex—some studies indicate PD-1 may restrain Treg activity, while others show it supports Treg stability—but in the tumor context, PD-1⁺ Tregs are generally associated with enhanced immunosuppressive function and poorer prognosis [56]. By utilizing these checkpoint pathways as “brakes” on immune activation, Tregs mirror their physiological role in preventing autoimmunity, but in cancer, this brake application overwhelmingly benefits the tumor.

Tregs restrain antigen-presenting cells via CTLA-4–dependent trans-endocytosis of CD80/CD86, reducing costimulation for CD28 on effector T cells [57]. In breast tumors, CTLA-4–high Tregs accumulate intratumorally (notably in TNBC), and their depletion or functional interference enhances CD8+ priming [18]. PD-1 and TIGIT further dampen effector activation (TIGIT competes with CD226), and TIGIT⁺/TIM-3⁺ Tregs display heightened suppressive capacity in human breast cancers [35,36].

Thus, checkpoint engagement enables Tregs to mimic physiological tolerance mechanisms, but in cancer, these brakes overwhelmingly suppress anti-tumor immunity.

(c) *IL-2 deprivation*. Tregs create a local growth factor sink that starves the effector lymphocytes. Notably, the high expression of CD25 (the IL-2 receptor α-chain) gives Tregs a competitive advantage in IL-2 uptake. They avidly consume IL-2 in the TME, a cytokine critical for T cell proliferation and survival, thereby depriving conventional T cells of this growth factor [29]. This IL-2 deprivation impairs the expansion and function of effector T cells.

(d) *Metabolic reprogramming*. IDO1 and IDO2 catalyze tryptophan degradation to kynurenine, which activates the aryl hydrocarbon receptor to promote Treg differentiation [58,59]. Treg ectonucleotidases (CD39/CD73) convert ATP→adenosine, activating A2A/A2B receptors on Teffs and NK cells to blunt cytotoxicity; adenosine-axis inhibitors are in IBC trials. IDO in tumor/immune compartments depletes tryptophan and generates kynurenine, an AhR ligand that promotes FOXP3 induction and Treg stability [59].; in breast cancer, IDO expression associates with Treg enrichment and adverse outcomes [60,61]. This pathway serves as both an independent prognostic indicator and a therapeutic target. Through these metabolic and enzymatic means, Tregs effectively suppress the fitness and proliferation of anti-tumor T cells in the tumor microenvironment [62].

Through these strategies, Tregs create a nutrient-deprived, metabolically suppressive TME that weakens effector responses, while reinforcing their own dominance.

(e) *Direct cytotoxicity*: One remarkable tactic is granzyme- and perforin-mediated cytolysis. Studies in mouse models have demonstrated that Treg-derived granzyme B and perforin are essential for the suppression of anti-tumor responses, and human tumor-infiltrating Tregs can likewise express these cytotoxic molecules [22,23,63]. Activated Tregs in tumors have been shown to upregulate Granzyme A/B and perforin, enabling them to directly kill susceptible target cells such as CD8+ T cells, B cells, or NK cells that they engage in close contact [22,23,63]. This contact-dependent cytotoxicity removes key effector cells from the immune arsenal. It has also been reported that Tregs can physically disrupt the formation of immunological synapses between DCs and T cells, and can even directly delete DCs via granzyme/perforin in specific contexts [53]., further ensuring that immune activation is kept in check. This direct cytotoxic activity ensures that activated effector cells are physically removed from the tumor milieu, representing a potent “last line” of immunosuppression.

(f) *Treg stability and plasticity in inflammatory breast TME.* FOXP3 function depends on acetylation (e.g., p300/Tip60) that stabilizes DNA binding and on balanced ubiquitination pathways [64].; inflammatory cues (IL-6, TNF) can destabilize FOXP3 and remodel the Treg epigenome [65]. Modulating these pathways offers opportunities to attenuate intratumoral Tregs while preserving systemic tolerance.

Under inflammatory cytokines (IL-12, IL-6, TNF) and strong TCR costimulation, Tregs can down-modulate FOXP3 and acquire Th1-like features (T-bet⁺, IFN-γ⁺ “ex-Tregs”), observed in human tumors including breast cancer [66]. While plasticity may transiently favor antitumor immunity, it can also yield unstable regulation and bystander tissue injury; thus, therapeutic designs should consider timing/dose to avoid systemic autoimmunity while leveraging intratumoral reprogramming.

In summary, in IBC, Tregs suppress antitumor immunity through coordinated checkpoint engagement, inhibitory cytokines, and metabolic rewiring (adenosine and IDO–kynurenine), while FOXP3 stability is reinforced by post-translational modification. These pathways are variably enriched across breast cancer subtypes and are associated with immune-excluded phenotypes and poorer outcomes. Importantly, Treg plasticity under inflammatory stress can transiently unlock effector programs, highlighting opportunities and risks for therapeutic reprogramming.

**Figure 1:**
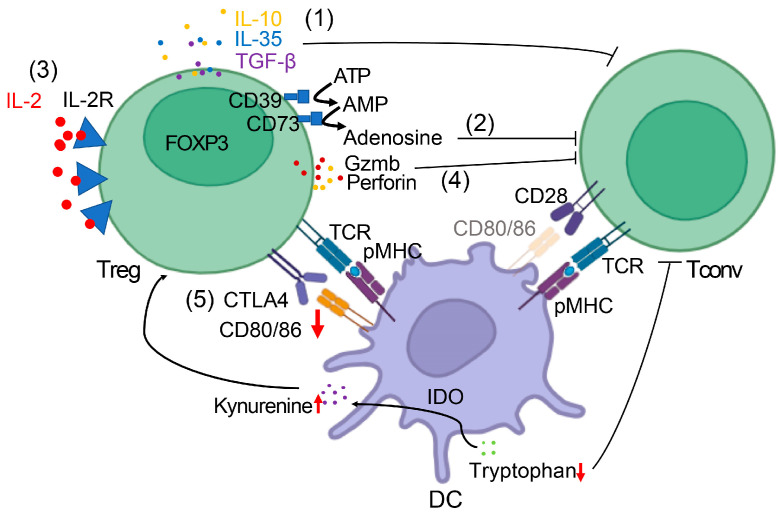
Mechanisms of Treg-Mediated Immunosuppression in Breast Cancer. Schematic illustration of the major pathways by which Tregs suppress anti-tumor immunity. Key elements include: (1) immunosuppressive cytokines (IL-10, TGF-β, IL-35) acting on effector T cells and dendritic cells; (2) CD39/CD73-mediated adenosine production; (3) IL-2 consumption depriving effector T cells; (4) granzyme/perforin-mediated cytotoxicity against effector lymphocytes; (5) checkpoint receptor interactions, e.g., CTLA-4 depleting CD80/86, which leads to overexpression of IDO in dendritic cells (DC); IDO catalyzes tryptophan degradation to kynurenine; kynurenine accumulation promotes Treg differentiation, while tryptophan reduction impairs the function of CD4+/CD8+ conventional T cells (Tconv). The figure depicts Tregs in direct interaction with Tconv and DC in the tumor microenvironment.

Figure 1 illustrates the major pathways by which Tregs suppress anti-tumor immunity. Together, these multifaceted mechanisms underscore why Tregs are considered *“enablers of immune evasion.”* By deploying immunosuppressive cytokines, engaging inhibitory checkpoints, usurping growth factors, altering metabolic cues, and even directly killing immune cells, tumor-associated Tregs create a profoundly tolerogenic microenvironment that protects the tumor from immune attack. Their pervasive influence in the tumor microenvironment is a significant barrier to effective anti-tumor immunity. Accordingly, the prevalence of Tregs in many cancers is a predictor of worse outcomes, and Tregs have become a focal point of cancer immunology research and an active target for new therapies [44]. Strategies to counteract Tregs, whether by depleting Tregs, reprogramming them, or blocking their suppressive pathways, are being actively explored as means to improve anti-tumor immune responses and tip the balance in favor of immune-mediated tumor control [67].

### 2.3. Pro-Tumorigenic Functions Beyond Immune Suppression

While Tregs are best known for dampening anti-tumor immunity, accumulating evidence indicates that they also support tumor progression through non-immunological mechanisms. These pro-tumorigenic functions include the promotion of angiogenesis, remodeling of the extracellular matrix (ECM), and facilitation of metastasis.

(a) *Promotion of angiogenesis and extracellular matrix remodeling***.** Tregs contribute to tumor progression by promoting angiogenesis and formation of new blood vessels that support tumor growth and metastasis. Angiogenesis requires the degradation of the vascular basement membrane and remodeling of the extracellular matrix (ECM) to allow endothelial cells to migrate into the surrounding tissue [68]. This process plays a critical role in cancer, as it facilitates the delivery of oxygen, nutrients, and growth factors, and enables tumor progression to distant organs [69]. Treg cells promote angiogenesis by secreting two key molecules: matrix metalloproteinases (MMPs) and vascular endothelial growth factor (VEGF). MMPs are a family of enzymes that degrade various components of the ECM and participate in the remodeling of basement membranes and the ECM [70]. They have been shown to contribute to tumorigenesis, progression, and angiogenesis in both early and late stages of cancer and are often upregulated in malignant tumors [71]. VEGF is a potent angiogenic factor and an essential growth factor for vascular endothelial cells, which form the inner lining of arteries, veins, and capillaries. In many tumors, VEGF is also upregulated and drives tumor angiogenesis [72]. Through the secretion of MMPs and induction of fibroblast activation, Tregs contribute to ECM breakdown and stromal remodeling. This not only facilitates local invasion but also generates a supportive niche for metastatic dissemination. Tregs function as indirect enablers of breast cancer invasiveness by influencing the stromal architecture. Thus, beyond immune regulation, Tregs actively shape the vascular microenvironment to sustain tumor growth.

(b) *Facilitation of metastasis.* Tregs accumulate in pre-metastatic niches and secrete factors that suppress local immune surveillance, paving the way for disseminated tumor cells to colonize distant organs. In murine breast cancer models, depletion of Tregs reduces lung metastases, reinforcing their role in metastatic seeding [73]. These findings suggest that Tregs extend their influence beyond the primary tumor, ensuring the survival and outgrowth of disseminated tumor cells.

Taken together, Tregs are not merely suppressors of anti-tumor immunity, but also active participants in tumor progression through angiogenesis, stromal remodeling, and metastatic facilitation. These functions highlight the dual role of Tregs as both guardians of tolerance and unwitting allies of cancer, making them attractive, yet challenging therapeutic targets. Recent reviews synthesize how Treg heterogeneity emerges from transcriptional, metabolic, and microenvironmental cues, and outline implications for therapeutic targeting in solid tumors [74,75].

## 3. Tregs as Prognostic Biomarkers in Breast Cancer

### 3.1. Clinical Evidence Linking Treg Abundance to Disease Outcomes

**Figure 2:**
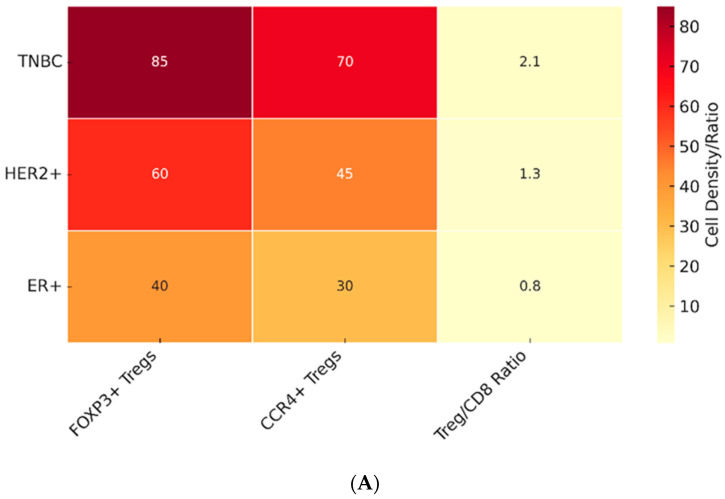

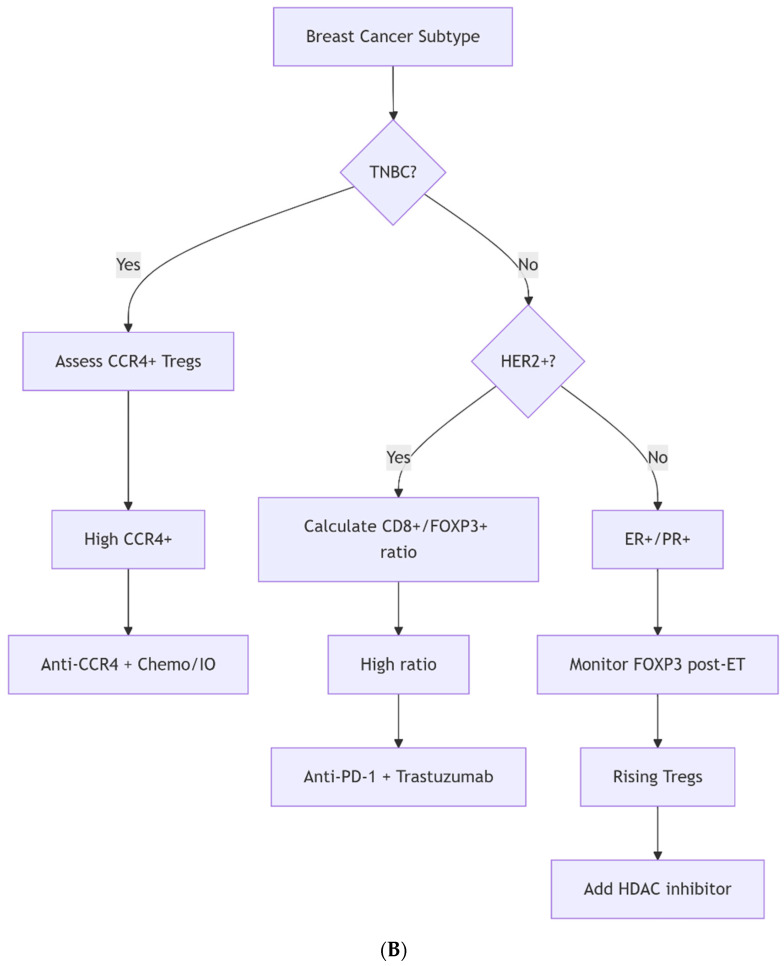
Treg-Based Precision Therapy Framework. (**A**) Treg Infiltration Heatmap Across Breast Cancer Subtypes. Heatmap summarizing relative FOXP3+ Treg infiltration in triple-negative breast cancer (TNBC), HER2-enriched, and ER⁺ subtypes. TNBC shows the highest density of FOXP3+ Tregs and lowest Treg/CD8 ratio, HER2⁺ tumors display intermediate levels with context-dependent prognostic impact, and ER⁺ tumors generally exhibit lower but clinically significant infiltration. Interpretation: TNBC showed the highest Treg infiltration (85 FOXP3+ cells/mm^2^) and the lowest Treg/CD8 ratio (2.1). ER+ tumors had lower but clinically significant Treg levels (40 cells/mm^2^). *Heatmap showing the median FOXP3+ Treg density across subtypes (TNBC > HER2+ > ER+). Data from 10 clinical cohorts (n = 2145). (**B**) Stratification Algorithm for Differential Therapy. Conceptual overlay illustrating how subtype-specific Treg infiltration patterns may inform treatment strategies. For example, Treg depletion combined with checkpoint inhibition may benefit TNBC, while HER2⁺ and ER⁺ subtypes may require context-specific therapeutic combinations. Data Sources for Figure 2A Heatmap. Subtype-specific FOXP3+ Treg densities (cells/mm^2^) were derived from a single-platform, automated multicolor IHC study quantifying stromal and intratumoral TILs across ER/PR+, HER2+, and TN cohorts [79]. For TNBC-focused and compartment-specific contexts, we referenced multiplex imaging that reports FOXP3+ counts per mm^2^ and their enrichment in CAF-S1–high tumors [80]. As an orthogonal validation in high-TIL, PD-L1–positive disease, we reported stromal FOXP3+ densities comparing TNBC and HR+ [81]. Historical ER− benchmarks with per mm^2^ quantitation are provided [77]. Absolute values vary by assay and compartment; therefore, medians were computed within a single platform [79]. and cross-checked qualitatively against the other datasets. HER2-Positive Breast Cancer. 1. Salgado et al. (2015)—NeoALTTO Trial [82]. 2. Loi et al. (2019)—PANACEA Trial [83]. 3. Emens et al. (2020)—KATE2 Trial [84]. Hormone Receptor-Positive (ER+/PR+). 1. Ali et al. (2014)—TransATAC Cohort [85]. 2. Smith et al. (2020)—PreOperative Endocrine Therapy for Individualized Care (POETIC) Trial [86]. Note: Absolute FOXP3+ densities (cells/mm^2^) have not been reported in these trials and would require dedicated multiplex IHC or spatial quantification studies. Cross-Subtype Meta-Analysis. Stanton et al. (2016)—81-Study Pooled Analysis [87]. Key: IO = immunotherapy; ET = endocrine therapy.

The prognostic significance of Treg infiltration in breast cancer has been the subject of extensive investigation, yielding results that vary according to molecular subtype and clinical context. In triple-negative breast cancer (TNBC), multiple cohort studies have demonstrated that high densities of FOXP3+ Tregs in the tumor stroma correlate with aggressive disease, early recurrence, and poor overall survival [76,77]. These findings are consistent with the immunogenic nature of TNBC, where the presence of suppressive immune subsets can negate otherwise favorable immune infiltration. In contrast, HER2-positive tumors present a more complex picture. Some studies indicate that Treg infiltration is associated with an enhanced response to trastuzumab and improved survival, possibly reflecting that Tregs accompany broader immune activation, including CD8+ cytotoxic T cells and NK cells [78]. As illustrated in Figure 2 The degree of Treg infiltration varies significantly across breast cancer subtypes, with the highest levels observed in TNBC and context-dependent effects in HER2^+^ disease.

Several Treg-related transcriptional signatures have been characterized in breast cancer and across solid tumors, providing insights into the immunosuppressive networks that shape the TME. CCR8-centered tumor-infiltrating Treg (TI-Treg) signatures highlight highly activated Tregs enriched in breast tumors, often co-expressing markers such as LAYN and MAGEH1 that correlate with poor prognosis [88,89]. Pan-cancer TI-Treg programs have validated these findings across multiple tumor types, emphasizing their conserved nature [88]. Inhibitory-receptor modules, encompassing checkpoints such as CTLA-4, LAG-3, TIM-3, and TIGIT, underscore the redundancy of suppressive pathways deployed by breast tumor Tregs [54,55]. Metabolic adaptations, particularly through adenosine-generating CD39/CD73 and the adenosine receptor ADORA2A, further reinforce Treg-mediated suppression in breast cancer [45,90]. Finally, prognostic signatures derived from bulk RNA-seq cohorts confirm that Treg-associated transcriptional programs predict survival outcomes and therapy responsiveness [91]. Collectively, these published modules replace earlier placeholder signatures and provide a more evidence-based framework to evaluate the role of Tregs in breast cancer prognosis and therapeutic resistance (Table 1).

Overall, the prognostic role of Tregs in breast cancer is not uniform but strongly influenced by spatial compartmentalization and molecular context. Multiple meta-analyses and systematic reviews have demonstrated that high FOXP3+ Treg infiltration is generally associated with worse survival in breast cancer patients, yet the magnitude and even direction of this effect varies [93,94,95,96]. In particular, stromal FOXP3+ enrichment has been consistently linked to adverse outcomes, whereas intratumoral FOXP3+ Tregs can exert neutral or even favorable associations in certain contexts such as TNBC, particularly when accompanied by robust CD8+ T cell infiltration or when inflammatory programs (e.g., IL-33/TGF-β signatures) are present [97,98]. These findings underscore that spatial localization, together with tumor subtype and stage, are critical determinants of the prognostic significance of Tregs. This evidence base highlights why interpretation of Treg density in breast cancer must be compartment-aware and integrated with other immune markers. Such complexity forms the basis of the so-called “Treg paradox” discussed in the following section.

### 3.2. Tregs in Different Breast Cancer Subtypes

While meta-analyses provide an overall picture of Treg abundance and outcomes in breast cancer, the impact of Tregs is not uniform across molecular subtypes. In TNBC, intratumoral FOXP3+ Tregs are sometimes associated with favorable prognosis when accompanied by dense CD8+ infiltrates, whereas stromal localization is more consistently adverse. In HER2-positive tumors, higher Treg densities often correlate with resistance to trastuzumab and worse survival. In hormone receptor–positive disease, particularly luminal B, Tregs are generally unfavorable, though their prognostic impact in luminal A cancers appears attenuated.

These patterns highlight that molecular subtype modifies the biological and clinical significance of Tregs. Subtype-specific immune signatures, integrating Treg localization with effector T cell markers, may therefore be more informative than bulk FOXP3+ counts alone.

### 3.3. The Treg Paradox: Context-Dependent Roles in Prognosis

Tregs are often found in abundance within the TME, where they efficiently infiltrate and adapt to local conditions. By dampening the activity of effector immune cells, Tregs help to create an immunosuppressive environment that allows tumors to evade immune surveillance. Consequently, a higher proportion of Tregs among tumor-infiltrating lymphocytes (TILs) is generally correlated with worse prognosis across various cancer types [99].

However, in HER2^+^ and ER^-^ breast cancers, elevated levels of FOXP3+ Tregs may coincide with enhanced survival, particularly when CD8+ T cells are also present. One study found that high levels of FOXP3+ TILs were significantly associated with improved survival in the HER2^+^/ER^+^ subgroup, especially when CD8+ cytotoxic T-cell infiltrates were also present [26]. This suggests potential cooperation between Tregs and CD8+ T cells in generating an effective anti-tumor response. Similarly, among HER2^+^/ER^-^ breast cancer patients with CD8+ T cell infiltration, those with high FOXP3+ TILs had a 52% higher survival probability than those with low FOXP3+ TILs. Nevertheless, in the core-basal subgroup, although high FOXP3+ lymphocyte infiltration was associated with better survival, the presence of CD8+ T cells appeared to be a significant prognostic factor. These findings highlight that high Treg infiltration, though commonly associated with poor outcomes, can signal a favorable prognosis in specific cancer subtypes, such as HER2^+^/ER^+^ and HER2^+^/ER^-^ breast cancers. Of note, single-cell and spatial transcriptomic studies in breast cancer now map Treg states and neighborhoods within specific subtypes (e.g., TNBC), linking immune architecture to clinical endpoints and therapy response [100,101,102,103].

### 3.4. Methodological Considerations in Treg Assessment

Some studies have suggested that FOXP3+ Treg infiltration can be used as a prognostic biomarker in cancer treatment. However, there were some discrepancies. Discrepancies often arise from the techniques used to identify Tregs. Immunohistochemistry (IHC) staining for FOXP3 is considered the gold standard for identifying Tregs in tissue sections. However, conventional T cells can transiently upregulate FOXP3 during activation, potentially leading to the overestimation of Tregs. Thus, IHC cannot distinguish true Tregs from transiently activated non-Tregs. Moreover, variations in the staining protocol, antibody specificity, and affinity can affect quantification. Flow cytometry can simultaneously analyze multiple markers, such as CD4, CD25, FOXP3, and others, including CD127 and CD45RA, to precisely identify true Tregs. However, the major limitation is that fresh or properly preserved single-cell suspensions are required, which limits its application to preserved solid tumor tissues. Marker selection and gating strategies can significantly influence Treg cell quantification. Quantitative analysis of gene expression of Treg markers (FOXP3, CTLA4, IL-2A) using next-generation sequencing or RT-qPCR provides an indirect assessment of Treg abundance. However, these methods may reflect both Treg infiltration and immune activation and may not distinguish between suppressive Tregs and non-suppressive conventional T cells.

Treg density varies substantially within different tumor regions, including the tumor core, invasive margins, and tertiary lymphoid structures. The uneven distribution of Tregs reflects differences in the local tumor microenvironment, such as hypoxia, necrosis, and immune cell infiltration. This spatial variability often makes the results from single-site biopsy unreliable and makes standardized quantification challenging. The definition of regions (core vs. margin) may differ between studies, further complicating the comparison. These technical differences and spatial heterogeneity of tumor tissues have led to inconsistent results in studies using Tregs as prognostic markers, necessitating standardized protocols and composite biomarker approaches.

Recent advances in digital pathology have greatly improved the accuracy and reproducibility of tumor-infiltrating lymphocyte (TIL) and regulatory T cell (Treg) measurements. Automated image analysis tools and artificial intelligence–assisted platforms reduce inter-observer variability that is frequently encountered in manual pathology review, providing more consistent quantification across cohorts. Notably, the Immuno-Oncology Biomarker Working Group for Breast Cancer has established consensus recommendations on the standardized assessment of TILs and Tregs, facilitating cross-study comparability and clinical translation [104]. In line with these efforts, Parra and colleagues have shown that multiplex immunofluorescence panels combined with image analysis yield robust cellular spatial and immune profiling in paraffin tumor tissues [105]. Together, these developments underscore the importance of incorporating digital pathology approaches into future biomarker studies to ensure robust, scalable, and clinically relevant measurements.

### 3.5. Composite Biomarkers for Improved Prognostication

Except for the aforementioned limitation of Treg assessment as a biomarker, measuring Treg abundance alone cannot reflect the complex interaction between different immune cells and immune cell-tumor cells, which are critical for clinical outcomes. Given the limitations of standalone Treg metrics, recent efforts have focused on integrative biomarkers: the Combination of Treg abundance with additional immune characteristics, including the presence and activity of CD8+ cytotoxic T cells, natural killer cells, and expression of immune checkpoint molecules. Composite biomarkers provide a comprehensive understanding of the tumor immune environment by integrating several related biological signals and identifying complex interactions within the TME.

(a) *CD8+/FOXP3+ Ratio:* The ratio of CD8+ cytotoxic T cells to Tregs has been developed as an important predictor of the potential immune response in cancer therapy. A high ratio (reflecting robust cytotoxic T cells relative to Tregs) predicts better responses to immunotherapy in metastatic TNBC [106]. Including this parameter in cancer treatment helps clinicians to better predict disease progression and decide therapeutic regimens than depending on Treg abundance data only.

(b) *Immune Contexture Scores:* In addition to immune cell number-based evaluation, data on cancer cell proliferation, inflammation, and cancer immune response can also be collected. For instance, cancers with high Treg numbers and low immune checkpoint molecule expression are likely to be less severe than those with both high Treg numbers and checkpoint molecule expression. Algorithms combining Tregs, CD8+ T cells, and PD-L1 expression (e.g., “Immunoscore”) are under validation for breast cancer [106,107]. This approach integrates Tregs abundance with multiple cancer immunology-related features altogether for a complete breast cancer assessment.

Furthermore, Treg-included composite biomarkers can be used to identify patient subgroups from the perspective of precision medicine. Patients with Treg-included composite biomarker profiles may be candidates for Treg-targeted therapy, while others may need to avoid excessive Treg-related treatment. Composite biomarkers not only increase the accuracy of breast cancer evaluation, but also benefit personalized therapy and eventually improve patient prognosis [87]. Utilizing composite biomarkers will allow Tregs to be considered in the clinical decision-making process, which has strong potential to improve breast cancer prognosis and therapy. There are several studies that need to be performed in the future: the standard protocol for identifying the Treg should be established, as this will make the Treg data from different sources comparable; most relevant components of composite biomarkers should be determined and the composite biomarkers must be tested for their clinical relevance in a large cohort of patients; and the guidelines for clinical therapy should be established based on the composite biomarkers.

## 4. Tregs and Therapeutic Responses

Therapeutic interventions for breast cancer profoundly influence Treg biology, with both conventional treatments and novel immunotherapies shaping the abundance and function of these suppressive cells. Understanding these dynamics is crucial for designing rational combination regimens that maximize anti-tumor immunity while minimizing immune-related toxicities.

### 4.1. Conventional Therapies and Treg Modulation

Because elevated Treg cells reduce immune responses against tumors, different conventional therapies have been developed to modulate Treg cells. Chemotherapy can enhance anti-tumor immunity by reducing the Treg cell population. Traditionally, the purpose of chemotherapy is to direct cytotoxicity to induce tumor death. Taxanes, a type of chemotherapeutic drug, contain docetaxel or paclitaxel and have been used to treat a variety of malignant cancers. In previous studies, docetaxel showed apparent anti-tumor effects by modulating immune or Treg cells. All three Treg subsets—CD4+CD45RA^−^CD25^hi^, CD4+CD45RA^+^CD25^lo^, and CD4+CD45RA^−^CD25^lo^—derived from PBMCs of NSCLC patients, were reduced following docetaxel treatment in vitro. Docetaxel-induced depletion of Treg cells can significantly prolong survival by improving anti-tumor immunity [108]. Other chemotherapeutic agents, such as anthracyclines, may promote immune activation despite their limited effects on Tregs. Another method of chemotherapy involves anthracyclines, specifically doxorubicin, which promotes immunogenic cell death. Studies have shown that doxorubicin treatment alone has no effect on peripheral blood T lymphocytes and Tregs in vivo or in vitro. However, CD4+ T cells were resistant to doxorubicin. They demonstrated more robust proliferation after pretreatment, as the expression of CD40 ligand and 4-1BB on the surface of CD4+ T cells was increased. This suggests that doxorubicin pretreatment in cancer patients may enhance anti-tumor immune responses by increasing antigen-specific CD4+ Th1 immune responses [109].

Low-dose (metronomic) cyclophosphamide has been shown to temporarily reduce circulating Tregs by over 40% in patients with metastatic breast cancer, and this reduction was associated with a stable increase in tumor-reactive T cells and improved clinical outcomes [110]. Moreover, taxanes such as paclitaxel have been observed to selectively reduce CD4+FOXP3+ Treg numbers and impair their function without affecting conventional effector T cells in breast cancer models [111]. In the neoadjuvant setting, regimens including docetaxel, doxorubicin, cyclophosphamide, or trastuzumab were also associated with decreased peripheral Treg numbers and improved CD8+/Treg ratios, particularly in HER2-positive cases, correlating with better overall survival [112]. Anthracycline-based neoadjuvant chemotherapy similarly reduced Treg abundance in tumor biopsies from TNBC patients, where lower Treg levels and higher CTL/Treg ratios were predictive of pathologic complete response (pCR) [113].

### 4.2. Immunotherapy and Treg-Targeted Strategies

Multiple forms of immunotherapy have been developed to target Tregs. A major approach involves the use of immune checkpoint inhibitors, including anti-PD-1/PD-L1 and anti-CTLA-4 (ipilimumab).

Immune checkpoint inhibitor therapies such as anti-PD-1/PD-L1 antibodies aim to restore anti-tumor immunity by reinvigorating CD8+ T cells. Programmed death-1 (PD-1) is a cell surface receptor that functions as a T-cell checkpoint and helps regulate T-cell exhaustion. Binding of PD-1 to its ligand, programmed death-ligand 1 (PD-L1), activates signaling pathways and inhibits T-cell activation. High PD-L1 expression on tumor cells mediates tumor immune escape; therefore, the development of anti-PD-1/PD-L1 antibodies has gained attention in cancer immunotherapy [99]. Although anti-PD-1/PD-L1 agents are designed to reinvigorate CD8+ T cells, these therapies may inadvertently expand Tregs in some patients, necessitating the use of combination approaches. Dodagatta-Marri et al. found that PD-1 blockade monotherapy could skew the Teff/Treg balance in favor of Tregs, thereby curtailing the anti-tumor effect. They observed that anti-PD-1 activated the TGF-β/Smad3 pathway in tumor cells, which could then promote tumor cell epithelial–mesenchymal transition (EMT) toward a myofibroblast phenotype and reduce antigen presentation activity to inhibit immune surveillance [114].

Similarly, anti-CTLA-4 immunotherapy enhanced anti-tumor responses by promoting Treg depletion through Fcγ receptor-mediated cytotoxicity. Anti-CTLA-4 (ipilimumab) targets the inhibitory receptor cytotoxic T-lymphocyte-associated protein 4 (CTLA-4), which is expressed at elevated levels in Foxp3+ Treg cells and transiently at low levels in conventional T cells upon activation. Anti-CTLA-4 antibodies have been developed to enhance anti-tumor responses, although the mechanism of the antibodies remains debated. Preclinical studies have demonstrated that the therapeutic efficacy of anti-CTLA-4 antibodies depends on their ability to engage Fcγ receptors (FcγRs) on immune effector cells. When anti-CTLA-4 antibodies bind to CTLA-4-expressing Tregs within the TME, their Fc regions interact with FcγRs on these effector cells, leading to selective depletion of Tregs through ADCC (antibody-dependent cellular cytotoxicity). This mechanism has been demonstrated by experiments showing that anti-CTLA-4 antibodies with effector-dead Fc regions or FcγR-deficient (FcγR^−^/^−^) mice fail to achieve anti-tumor effects, indicating that Treg depletion is important for therapeutic benefit. However, a recent study on the function of anti-CTLA-4 antibodies suggested that the majority of efficacy is a result of myeloid compartment reprogramming through FcγR interactions [115].

### 4.3. Emerging Treg-Specific Therapeutic Approaches

In breast cancers, CCR8 is consistently upregulated in tumor-resident FOXP3+ Tregs compared to those in normal tissue or blood, and patients with higher CCR8⁺ Treg infiltration tend to have poorer survival [116,117,118]. Bulk and single-cell RNA sequencing data reinforce that CCR8 expression correlates tightly with FOXP3, suppressive phenotypes, and immune checkpoint gene signatures. Several engineered IL-2 molecules are now demonstrating preferential expansion of effector T cells with limited Treg stimulation. For example, sumIL-2-Fc (mutated IL-2 with reduced CD25 binding) shows enhanced CD8+ versus Treg responses and better tumor control in murine models [119]. Long-acting “superkines” similarly shift the balance toward cytotoxic effectors [120]. Additionally, cis-targeting of IL-2 to CD8+ T cells improves antitumor activity compared to unmodified IL-2 by avoiding widespread Treg expansion [121]. Mutant IL-2 designs with altered receptor affinity further support this preference in preclinical models [122]. In addition, checkpoint targets beyond PD-1 and CTLA-4 are advancing. TIGIT blockade has been shown to restore effector T cell function and modulate Treg activity, with preclinical studies demonstrating that TIGIT signaling promotes a highly suppressive Treg phenotype [33,35,36]. OX40 agonists enhance effector T-cell proliferation/survival and can diminish Treg-mediated suppression, as shown in first-in-human trials of MEDI0562 and MOXR0916 [123,124].

Anti-CCR4 antibodies, such as mogamulizumab, also represent an emerging agent for depleting Tregs and enhancing anti-tumor immunity. Mogamulizumab is the first approved monoclonal antibody that targets CC chemokine receptor 4 (CCR4), which is primarily expressed on Tregs and helper T cells, where it functions to induce the homing of leukocytes to sites of inflammation. Mogamulizumab is an anti-CCR4 antibody with a defucosylated Fc region, which enhances ADCC. In addition, mogamulizumab depletes CCR4+ Tregs, potentially evoking anti-tumor immune responses via autologous effector cells [125].

Another emerging agent, CD25-directed therapy, such as denileukin diftitox (IL-2-diphtheria toxin fusion), offers a means of targeting CD25+ Tregs but may also cause loss of effector T cells. Denileukin diftitox is a fusion protein composed of interleukin (IL)-2 and diphtheria toxin. It selectively targets cells expressing the CD25 component of the IL-2 receptor, and its diphtheria toxin component inhibits protein synthesis, leading to apoptosis [126]. A study found that after continuous treatment with 5 nM denileukin diftitox for 72 h, there was a significant reduction in the CD4+CD25^high^ human Treg population. The study also found that a single intraperitoneal injection of denileukin diftitox was sufficient to decrease the frequency of Treg cells in the spleen, peripheral blood, and bone marrow of treated mice [127]. However, a drawback of CD25-directed global intervention is that, while it achieves transient Treg depletion, it also depletes CD25+ effector T cells that upregulate CD25 following activation [128].

Bispecific antibody platforms represent another novel avenue, linking tumor antigen recognition to Treg depletion or functional blockade, thereby combining specificity with immunomodulation [129]. Although primarily developed in autoimmunity, CAR-Tregs demonstrate the feasibility of engineering suppressive cells; inversely, harnessing CAR approaches against Tregs in tumors may provide novel avenues for selective elimination [34]. Nanoparticle-based strategies have also been used to deliver cytotoxic payloads or siRNA directly to intratumoral Tregs, achieving local suppression of Treg activity with limited systemic exposure [130,131,132,133].

Beyond monotherapy approaches, rational combinations of Treg modulation with established cancer treatments are increasingly investigated. Radiotherapy can trigger antigen release and local inflammation, but this effect is often curtailed by rapid Treg recruitment [134]. Preclinical models show that combining radiotherapy with Treg depletion or functional inhibition enhances antigen-specific CD8+ T cell responses and prolongs tumor control [135]. For chemotherapy, low-dose/metronomic cyclophosphamide can transiently reduce or inhibit Tregs and is commonly paired with vaccines, endocrine therapy, or checkpoint blockade [136,137,138,139].

**Figure 3:**
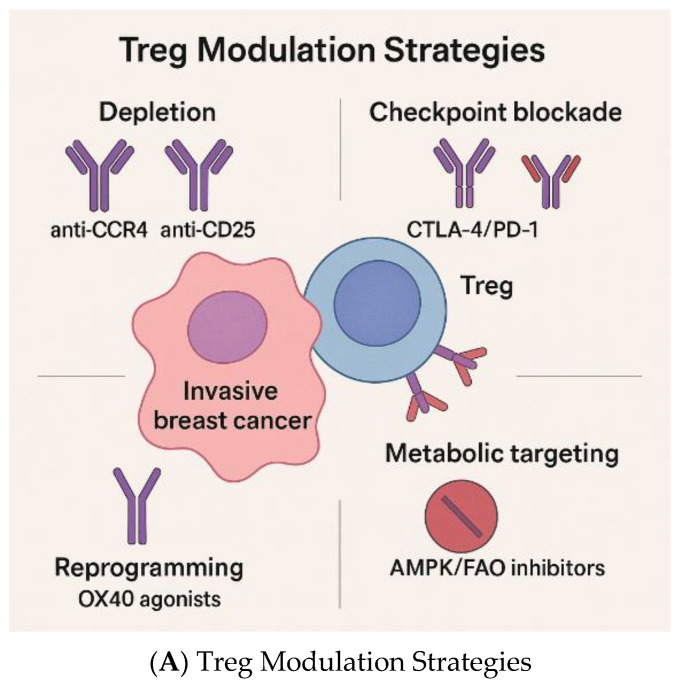

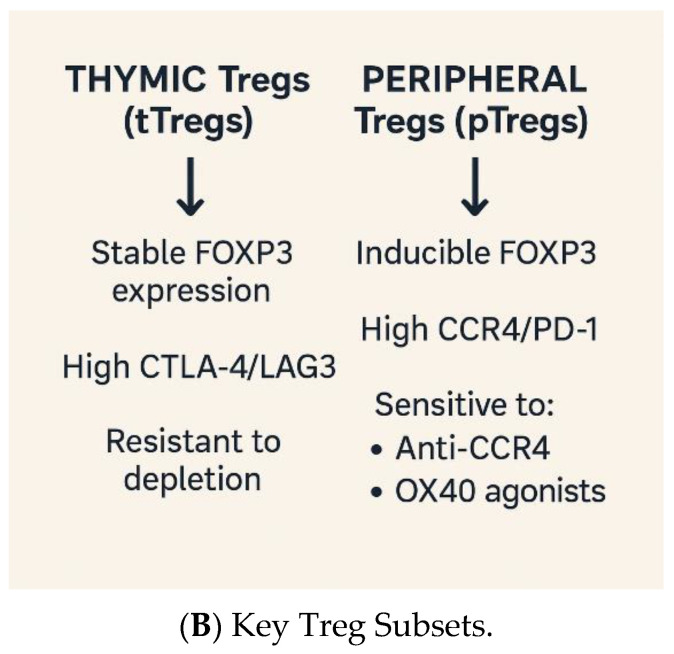
Precision Framework for Treg-Targeted Therapies in Breast Cancer. (**A**) A conceptual workflow diagram illustrating how Treg biology can be translated into precision medicine strategies. The figure should include: (1) patient stratification using composite biomarkers (CD8+/Treg ratio, FOXP3 expression, CCR4/CCR8 profiles); (2) subtype-specific strategies (e.g., Treg depletion combined with PD-1 blockade in TNBC; immune activation monitoring in HER2⁺); (3) therapeutic modalities (anti-CCR4 antibodies, low-dose cyclophosphamide, checkpoint inhibitors, metabolic modulators, OX40 agonists); and (4) anticipated outcomes (enhanced effector function, improved immunotherapy response, reduced recurrence). The figure should visually emphasize a balanced approach that maximizes tumor control while minimizing systemic autoimmunity. (**B**) Two major Treg subsets: Thymic Tregs (tTreg) and peripheral Tregs (pTreg). Key difference: pTregs are the primary target in breast cancer TME.

The various Treg-targeting therapeutic strategies are summarized in Figure 3. Table 2 summarizes the therapeutic applications that directly or functionally modulate Tregs within solid tumors, including breast cancer. Approaches include selective Treg depletion (CD25 and CCR8), checkpoint blockade with Fc-mediated Treg removal (anti-CTLA-4), metabolic/enzymatic pathways that sustain Tregs (CD73/adenosine, IDO1), and co-stimulatory agonists (GITR). Evidence ranges from mechanistic preclinical studies to early phase trials; large, randomized data remain sparse for breast cancer specifically, so the table emphasizes Treg-relevant mechanisms and representative studies in solid tumors, where breast-applicable biology is the strongest. (Key supporting studies include [28,140,141,142,143,144,145,146]).

### 4.4. Targeting Tregs in Clinical Trials

Prospective neoadjuvant datasets demonstrate that the immune contexture, particularly the CD8+/FOXP3+ (Treg) balance, is associated with pCR. In HER2-negative Gepar cohorts, higher baseline stromal TILs predicted significantly higher pCR under anthracycline/taxane chemotherapy [153]. Independent neoadjuvant series report that a higher CD8+/FOXP3+ ratio, at baseline or increasing on therapy, correlates with pCR and favorable outcomes [154,155]. In GeparNuevo (chemotherapy ± durvalumab), immune-inflamed tumors with higher TILs and IFN-γ-responsive transcripts achieved higher pCR, and translational correlative-linked effector-skewed CD8+/Treg balance with benefit [156]. Within I-SPY2, neoadjuvant PD-(L)1-containing arms showed that immune infiltrates and multi-platform biomarkers, when interpreted alongside FOXP3/Treg measures, enrich for pCR, with later analyses indicating that a composite immune contexture outperforms single markers [157]. Table 3 lists some ongoing or completed clinical trials that target Tregs in breast cancer.

### 4.5. Challenges in Treg-Targeted Therapy

Despite the potential of Treg-targeted therapy in cancer treatment, systematic Treg depletion can trigger autoimmunity, which can have unintended, harmful impacts on patients. Treg-mediated immunosuppression helps maintain self-tolerance against these diseases. When Treg cells are depleted and this system fails to operate properly, the immune system can react against self-antigens and destroy host organs, thereby causing autoimmune diseases including thyroiditis and colitis [158].

In response to Treg-mediated therapy, tumors may adopt compensatory mechanisms by upregulating alternative immunosuppressive pathways such as those mediated by IDO and myeloid-derived suppressor cells (MDSCs). IDO is expressed in many human cancers, and high IDO expression is associated with tumor metastasis. In cancer, IDO can be either expressed directly by the tumor cells themselves or indirectly induced in host antigen-presenting cells in the presence of the tumor. By driving IDO overexpression, tumors can create an immunosuppressive microenvironment that blocks the anti-tumor immune response. IDO inhibits the activation of effector T cells by depleting the essential amino acid tryptophan and promoting the activation of Foxp3+ Tregs through kynurenine production. In this study, we developed a B16 melanoma model overexpressing IDO to delineate the mechanisms of IDO-induced immunosuppression in tumor cells. When implanted into mice, B16-IDO tumors exhibited aggressive tumor growth and resistance to T cell-targeting immunotherapies. This process was associated with marked recruitment of MDSCs, as well as pathologically activated neutrophils and monocytes with potent immunosuppressive activity. A similar correlation between IDO expression and MDSC infiltration was observed in human melanoma samples and other animal tumor models that express high levels of IDO [159].

### 4.6. The Next Steps

The next decade will be critical in determining whether Treg-targeted strategies can meaningfully improve the outcomes of patients with breast cancer. Key priorities include refining biomarkers to distinguish immunosuppressive intratumoral Tregs from their systemic counterparts essential for immune tolerance. Composite measures such as the CD8+/FOXP3+ ratio, spatial immune context, and tertiary lymphoid structure density are promising tools for guiding patient selection.

Therapeutically, early phase studies are needed to adapt strategies proven in other cancers, such as CCR4 or CCR8 antibodies, CD25-directed toxins, and PI3Kδ inhibitors, to breast cancer–specific settings. Rational combinations are particularly compelling; pairing Treg modulation with checkpoint blockade, HER2-targeted agents, or chemotherapy may enhance efficacy without excessive toxicity. Importantly, subtype-specific approaches will be required; for instance, Treg depletion may benefit triple-negative disease, but could have divergent effects in hormone receptor–positive or HER2⁺ subtypes.

Finally, safety considerations must be at the forefront. Global Treg ablation risks autoimmunity, mandating approaches that either selectively target tumor-resident Tregs or transiently reprogram their suppressive function. The embedding of correlative immune monitoring in ongoing breast cancer trials will accelerate this process. If these challenges can be addressed, Treg-directed therapies can be developed, and hold the potential to expand the armamentarium of immunotherapy in breast cancer and advance toward precision, immune-guided care.

## 5. Future Directions

The intricate role of Tregs in breast cancer highlights several important avenues for future research and clinical translation. These directions emphasize precision targeting, rational combinations, advanced modeling, and integration into clinical trial designs.

### 5.1. Precision Modulation and Biomarker-Guided Approaches

Future strategies will likely rely on biomarkers to identify patients most likely to benefit from Treg-targeted therapies. Advances in single-cell RNA sequencing, CITE-seq, and spatial transcriptomics are uncovering Treg heterogeneity and context-specific vulnerabilities [160]. Candidate markers such as CCR8, TIGIT, and Helios are being evaluated as means to selectively target intratumoral Tregs while preserving systemic tolerance. Composite biomarkers, including the CD8+/Treg ratio, tertiary lymphoid structure (TLS) density, and interferon-related gene signatures, require prospective validation as predictors of clinical response.

### 5.2. Rational Combinatorial Strategies

Because Treg suppression operates alongside multiple immunoregulatory circuits, future therapies are unlikely to rely on single agents. Rational combination regimens may include checkpoint blockade with selective Treg depletion (e.g., anti-PD-1 plus anti-CCR8), epigenetic modulation combined with immunotherapy, or metabolic interventions targeting adenosine signaling and fatty acid oxidation. These approaches aim to destabilize Tregs while simultaneously reinforcing the persistence of effector T cells. Subtype-specific strategies are also critical; TNBC may benefit from dual checkpoint plus Treg modulation, HER2⁺ tumors from integration with HER2-targeted antibodies, and ER⁺ tumors from tailored combinations with endocrine therapy.

### 5.3. Advanced Preclinical Models and Translational Platforms

Clinical progress will depend on improved preclinical modeling. Humanized mouse models that incorporate patient-derived tumors and autologous immune repertoires enable evaluation of Treg-targeting agents in relevant contexts. Organoid-based immune co-cultures are emerging as rapid, flexible systems for testing Treg–effector–tumor interactions and drug responses. Integrative multi-omics, including spatial transcriptomics, proteomics, and epigenomics, will further clarify Treg plasticity and crosstalk with the stromal and myeloid compartments. Artificial intelligence-driven digital pathology offers another tool for standardizing the quantification of Tregs, CD8+ T cells, and TLS in clinical samples.

### 5.4. Implementation in Clinical Trials

Embedding Treg-focused hypotheses into prospective breast cancer trials is essential for their translation. This includes incorporating comprehensive baseline and on-treatment immune profiling, biomarker-driven cohort stratification, harmonized correlative endpoints (such as scRNA-seq, multiplex IHC, and spatial transcriptomics), and careful monitoring of immune-related adverse events. Adaptive platform trials and multi-institutional consortia may accelerate progress by testing multiple Treg-targeted strategies in parallel and by rapidly integrating correlative science.

Overall, the future of Treg-directed therapy for breast cancer will depend on precisely modulating suppression within tumors while preserving systemic tolerance, leveraging new biomarkers and technologies to inform patient selection, and embedding these insights into innovative trial designs.

### 5.5. Artificial Intelligence (AI)—Driven Approaches

The use of AI in biomedicine is rapidly increasing owing to the low-cost, high-reward nature of AI prediction. In therapeutics, prognosis, and diagnosis of invasive breast cancers, this is highlighted by the ability of AI to predict multi-billion parameter models, outperforming classic statistical models, and sometimes even human conclusions. Below is a brief review of the current use of AI in invasive breast cancer therapeutics, prognosis, and diagnosis, as well as its potential use in Treg cell research.

(a) *Diagnosis*—One of the earliest uses of AI, the diagnosis of invasive breast cancer, benefits from the granular attention that AI pays to both image and text inputs. Reabal Najjar found that AI usage in medical imaging analysis has positively reshaped the field of radiology [161]. Najar also found that AI use in analyzing common medical imaging techniques (such as MRI, PET scans, etc.) decreased diagnostic interpretation times from 11.2 days to 2.7 days on average and integrated patient history into image analysis, improving diagnostic confidence [161].

AI has also been used to identify new biomarkers using “omics” data. In 2025, Alum highlighted how AI has been used to find patterns in large datasets, allowing for better judgements regarding patient reactions and enhancing precision medicine [162]. This can be especially important for invasive breast cancers such as TNBC, which has a distinct lack of response to treatments, and can benefit greatly from an increase in biomarker targeting.

Another important use of AI in diagnostics is its ability to assess an exceptionally large number of parameters and draw conclusions from historical data. As the context window sizes increase, this strength is further highlighted as AI will be able to consider more history. Furthermore, with modern AI systems, hundreds of billions of parameters can be considered when making a prediction, allowing AI to assist in a patient’s diagnosis and prognosis by conducting a thorough review of their history and any tests and scans performed during their lifetime.

(b) *Prognosis*—The use of AI in prognosis is a new and ethically contentious topic. While some studies have shown that AI usage in predicting patient survival is accurate [163], the ethical aspects of using AI in such a way are still variable. Overall, AI predictions of patient survival, cancer risk, and metastasis have been shown to be accurate [163], especially when combined with other statistical techniques to provide clinicians with a thorough understanding of disease progression.

AI has also been shown to be useful in predicting treatment responses, which enhances both the therapeutic aspects and prognosis of patient care [163]. Further studies could be conducted to understand how accurate AI could be used to predict treatment responses, and how this could be used to create a personalized treatment plan for patients.

(c) *Therapeutics*—A 2022 review article explored the ways in which AI can be used to help discover anti-cancer targets within human cell lines, allowing for a more thorough exploration of the complex world of cell biology [164]. As previously mentioned, AI is capable of reviewing hundreds of billions of parameters at once and forming powerful connections that might be overlooked by a human scientist. This allows for a quickened approach for drug identification and testing, which broadens the horizon of available drugs for precision medicine. Such an approach is extremely important for invasive breast cancers, especially TNBC, and requires further drug discovery. The function of Tregs within this can also be further explored, as Tregs are known to be involved in a series of complex inter-cellular regulation pathways; thus, benefitting from AI’s ability to track billions of parameters.

In addition to drug discovery, AI-assisted therapeutic planning has been used in radiotherapy to precisely map tumor borders. This is possible because of AI’s attention to granular detail, something that human technicians might not be able to complete as effectively. A lab at the Winship Cancer Institute has been experimenting with the use of AI in focusing radiotherapy to limit the amount of collateral damage caused by the exposure of healthy cells to radiation [K. Ggoldenberg (2024). How AI is Changing Cancer Medicine: The Quiet Revolution. *Emory Winship Cancer Institute*].

Overall, the use of AI in cancer treatment is expensive, and with technology increasing in strength every few months, it is possible that AI assistance in drug development and distribution will become the norm. In Treg-focused invasive breast cancer research, it is extremely important to discover novel molecular targets, pathways, and intercellular relationships. However, there are many ethical considerations when sharing sensitive patient information with an AI. If patient anonymity and consent are not thoroughly tracked throughout the process, HIPPA violations may occur, particularly if the data are saved in a database used for AI training. Additionally, there is always the risk of hallucinations when an AI is not properly prompted or given context. This means that expert human guidance is always necessary when using AI as an assistive technology. It is always important to remember that AI cannot invent anything “new” on its own but simply finds connections and repeats concepts that it learned during training- likening to the analogy of the “stochastic parrot.”

## 6. Limitations

While substantial progress has been made in understanding the role of Tregs in breast cancer, several limitations constrain the current knowledge and translation. First, technical variability in Treg identification (IHC, flow cytometry, and gene expression profiling) complicates cross-study comparisons and may inflate or underestimate their true abundance. Second, spatial heterogeneity within tumors, for example, ore vs. margin vs. TLS, limits the reliability of single-biopsy analyses. Third, the functional plasticity of FOXP3+ cells means that not all FOXP3+ TILs are bona fide suppressive Tregs, and distinguishing between these subsets remains a challenge. Fourth, most mechanistic insights are derived from murine models or ex vivo assays, which may not fully recapitulate the immune dynamics of human breast tumors. Finally, therapeutic strategies targeting Tregs risk systemic autoimmunity and may be undermined by compensatory immunosuppressive pathways, such as IDO or MDSCs. Addressing these challenges is critical for the safe and effective translation of Treg-targeted interventions into clinical practice.

## 7. Clinical Implications

Regulatory T cells (Tregs) in breast cancer represent both a prognostic marker and a therapeutic target with direct implications for clinical management. High Treg infiltration is generally associated with poor outcomes, especially in triple-negative breast cancer (TNBC), where it can negate the otherwise favorable effects of tumor-infiltrating lymphocytes. Conversely, in some HER2^+^ and ER^−^ subtypes, Treg abundance may coincide with improved outcomes when accompanied by robust CD8+ T-cell infiltration, underscoring the need for context-specific interpretation.

From a therapeutic perspective, the modulation of Tregs could enhance existing treatments. Conventional chemotherapies, such as taxanes and low-dose cyclophosphamide, partially reduce Treg populations, contributing to an improved immune balance. Immunotherapies, including PD-1/PD-L1 and CTLA-4 checkpoint inhibitors, may either deplete or expand Tregs, depending on the clinical setting, highlighting the importance of rational combination strategies. Emerging agents, such as anti-CCR4 (mogamulizumab), CD25-directed fusion proteins, and CCR8- or TIGIT-targeted therapies, are under clinical evaluation and may selectively modulate intratumoral Tregs with less systemic toxicity.

In clinical practice, composite biomarkers such as the CD8+/FOXP3+ ratio, tertiary lymphoid structure density, and spatial immune context scores provide more reliable prognostic and predictive values than Treg abundance alone. These integrative measures could guide patient selection for Treg-targeted approaches and inform rational combinations with HER2-targeted therapy, endocrine therapy, or immune checkpoint blockade.

Finally, safety remains the central consideration. Global Treg depletion risks autoimmunity, and tumors may upregulate compensatory suppressive pathways (e.g., IDO, MDSCs). Therefore, clinical translation requires the precise modulation of tumor-specific Tregs while preserving systemic tolerance. Embedding correlative immune analyses in clinical trials will accelerate the identification of patient subgroups that are most likely to benefit. Over the next decade, Treg-directed interventions have the potential to shift breast cancer management toward biomarker-driven immune-based precision medicine.

## 8. Conclusions

Regulatory T cells are central players in the breast tumor environment, where they shape prognosis, influence treatment responses, and represent both obstacles and opportunities for therapy. Evidence to date underscores their dual nature—preserving immune balance under normal conditions but enabling tumor immune escape in cancer. Advances in single-cell technologies, spatial profiling, and integrative biomarkers now allow a deeper understanding of these cells and their heterogeneity across breast cancer subtypes. Translational progress will depend on approaches that selectively target tumor-promoting regulatory T cells while maintaining systemic tolerance. With carefully designed strategies, including rational therapeutic combinations and precision biomarker guidance, regulatory T cell modulation has the potential to transform breast cancer care and improve patient outcomes.

## Figures and Tables

**Table 1:** Published Treg-Related Signatures and Modules in Breast Cancer.

Signature/Module	Representative Genes	Evidence in Breast Cancer/Outcome Link	Key References
Breast tumor-infiltrating Treg (TI-Treg) marker set (CCR8-centered)	CCR8, FOXP3, IL2RA (CD25), CTLA4, ICOS, TIGIT; tissue/TI-Treg markers LAYN, MAGEH1	Highly activated TI-Tregs in breast tumors selectively upregulate CCR8; co-expression of LAYN/MAGEH1/CCR8 correlates with poor prognosis.	[88,89].
Pan-cancer TI-Treg (TITR) signature	CCR8, IL1R2, LAYN, MAGEH1, CTLA4, ICOS, TNFRSF1B (TNFR2)	Conserved TI-Treg program identified across human tumors, validated computationally and functionally, with breast cancer cohorts included in cross-tumor analysis.	[88,89].
Inhibitory-receptor (checkpoint) module (“Immunosuppressive”)	CTLA4, LAG3, HAVCR2 (TIM-3), TIGIT, ICOS; often co-expressed with CCR8	Human TI-Tregs upregulate multiple checkpoints, including TIM-3 and LAG-3, as part of the activated tumor-Treg phenotype; linked to suppression in breast cancer and other solid tumors.	[54,55].
Adenosinergic (“Metabolic-Treg”) module	ENTPD1 (CD39), NT5E (CD73), ADORA2A	CD39/CD73+ Tregs generate adenosine, suppressing anti-tumor immunity. In breast cancer, CD73+ γδ Tregs and CD39/CD73-high Tregs correlate with poor prognosis.	[29,92].
Breast cancer Treg-associated prognostic signatures (data-driven)	Study-defined (e.g., 6-gene Treg-associated prognostic signature)	Prognostic signatures derived from TCGA and other BC cohorts link Treg biology to survival and therapy sensitivity.	[91].

Footnote: These signatures represent reproducible gene sets or functional modules identified in previous studies. The genes listed are representative examples.

**Table 2:** Treg-modulating agents/strategies in breast cancer and other solid tumors (representative evidence).

Mechanism/Strategy	Example Agent(s)	How It Relates to Tregs	Representative Evidence (Preclinical/Clinical)	Key References
CTLA-4 blockade with Fc-effector activity	Ipilimumab; Fc-optimized anti-CTLA-4 variants	Preferential intratumoral Treg depletion via FcγR-mediated effector functions; contributes to efficacy	Mouse and humanized models show Treg depletion augments anti-tumor immunity; clinical correlative data support Fc-dependence	[28,141,147].
CD25 (IL-2Rα)-targeted Treg depletion (non-IL-2-blocking)	RG6292/vopikitug (afucosylated anti-CD25)	Selective Treg depletion while preserving IL-2 signaling on Teff cells	Potent Treg depletion and synergy with ICB in preclinical models; early clinical reports show on-target Treg reduction	[142,148,149].
CCR8-directed depletion of tumor-resident Tregs	BMS-986340, DKY709, BAY 3375968	CCR8 is enriched on intratumoral Tregs; antibodies aim to deplete these cells	Preclinical: CCR8 mAbs deplete Tregs and boost CD8 responses; Clinical: first-in-human CCR8 mAb BMS-986340 ongoing (NCT04895709)	[150].; NCT04895709 (BMS-986340).
CD73 (ecto-5′-nucleotidase) blockade	Oleclumab (MEDI9447)	Lowers adenosine production that sustains Tregs and suppresses Teff cells	First-in-human safety/pharmacodynamic data; combinations under study in solid tumors	[140].
Adenosine receptor antagonists (A2A/A2B)	Ciforadenant (CPI-444); Etrumadenant (AB928)	Block adenosine signaling that promotes Treg function and inhibits effector T cells	Preclinical CPI-444 restores T-cell function and synergizes with ICB; etrumadenant shows acceptable PK/PD and early clinical safety	[143,151].; Seitz L et al., *Invest New Drugs* 2019 (AB928 phase-1 HV).
IDO1 inhibition (tryptophan–kynurenine axis)	Epacadostat	Aims to limit tolerogenic DC and Treg-supportive metabolism	Phase III ECHO-301/KEYNOTE-252 (melanoma) negative for efficacy with pembrolizumab; concept under reevaluation	[144].
GITR agonism	TRX518; MK-4166; BMS-986156	Can attenuate Treg suppressive function and costimulate Teff	Early-phase trials show pharmacodynamic effects (Treg reduction/activation markers) with modest single-agent activity; combinations under study	[145,152].
TGF-β pathway blockade/trap-PD-L1 fusion	Galunisertib; Bintrafusp alfa	TGF-β supports immune exclusion and Treg-dominant TMEs; blockade may relieve suppression	Urothelial cancer study linked TGF-β signaling with T-cell exclusion and resistance to PD-L1; multiple combo trials in solid tumors	[146].

Footnote: Most clinical data are pan-solid tumors; breast-specific, Treg-focused readouts remain limited. The adenosine axis (CD73/A2A/A2B) and selective Treg-depleting antibodies (CD25 and CCR8) are the most explicit Treg-targeted approaches in development.

**Table 3:** Ongoing or completed clinical trials targeting Tregs in breast cancer.

NCT ID	Intervention	Target	Phase	Status	Notes/Outcomes
NCT04895709	BMS-986340	CCR8	I/II	Recruiting	Selective depletion of intratumoral Tregs; biomarker analyses ongoing.
NCT04158583/NCT04642365	RG6292 (vopikitug)	CD25	I	Terminated	Demonstrated both peripheral and intratumoral Treg depletion; however, clinical efficacy was limited in overcoming resistance.
NCT02281409	Mogamulizumab (KW-0761)	CCR4	I/II	Completed	Achieved Treg reduction; safety acceptable; limited breast cancer-specific benefit observed.
NCT03719326	Etrumadenant (AB928) ± Pembrolizumab	A2A/A2B	I/Ib	Completed	Adenosine pathway blockade reduced Treg-mediated suppression; combination approach under further evaluation.

Footnote: This table includes clinical trials with direct or selective Treg-targeting strategies in breast cancer (CCR8, CD25, CCR4, A2A/A2B). Broader immunomodulatory approaches that may indirectly affect Tregs (e.g., PD-L1/TGF-β traps such as Bintrafusp alfa) are excluded to maintain focus on Treg-directed interventions.

## Glossary

Glossary of Key Terms
Adenosine Pathway (CD39/CD73) An enzymatic cascade on Tregs that converts ATP into immunosuppressive adenosine.
Angiogenesis Formation of new blood vessels is promoted by Treg-derived VEGF and MMPs, supporting tumor growth and metastasis.
CCL22/CCR4 Axis Chemokine–receptor signaling pathway that recruits CCR4⁺ Tregs into the tumor microenvironment.
CD25 (IL-2 receptor α-chain) A High-affinity IL-2 receptor subunit constitutively expressed on Tregs; allows IL-2 consumption and effector T-cell starvation.
CD36 A fatty acid transporter upregulated in intratumoral Tregs, supporting metabolic adaptation and suppressive functions.
CD8+ T Cells Cytotoxic lymphocytes that kill cancer cells directly via perforin and granzyme release.
Checkpoint Inhibitors Immunotherapies targeting PD-1/PD-L1 or CTLA-4 that restore effector T cell function.
Composite Biomarkers Integrated immune metrics (e.g., CD8+/FOXP3+ ratio and immune context scores) predict outcomes more accurately than Tregs alone.
CTLA-4 Inhibitory checkpoint receptor on Tregs; competes with CD28 for CD80/CD86 binding, reducing T-cell activation.
Denileukin Diftitox Fusion protein combining IL-2 and diphtheria toxin; depletes CD25+ Tregs but may also affect activated effector T cells.
Epithelial–Mesenchymal Transition (EMT) A biological process in which epithelial tumor cells acquire invasive mesenchymal traits; promoted by TGF-β from Tregs.
FOXP3 Master transcription factor defining Treg lineage and function.
Granzyme/Perforin Cytotoxic proteins secreted by Tregs to directly kill effector immune cells.
IDO (Indoleamine 2,3-dioxygenase) This enzyme depletes tryptophan and produces immunosuppressive metabolites that promote Treg activity.
Immune Contexture Score Composite measure of immune infiltrates, proliferation, and checkpoint expression that refines prognosis.
LAG-3, TIM-3, TIGIT Inhibitory checkpoint receptors on Tregs and effector T cells contribute to this suppression.
Metronomic Chemotherapy Low-dose, continuous chemotherapy (e.g., cyclophosphamide) that selectively reduces Tregs while sparing effector T cells.
Mogamulizumab Monoclonal antibody targeting CCR4; selectively depletes CCR4⁺ Tregs.
PGE2 (Prostaglandin E2) Lipid mediators produced in tumors that induce FOXP3 expression and enhance Treg activity.
PD-1/PD-L1 Checkpoint receptor–ligand pair suppressing T cell activity and sustaining Treg function in tumors.
Regulatory T Cells (Tregs) CD4+CD25+FOXP3+ lymphocytes are essential for immune tolerance but are co-opted by tumors to suppress immunity.
Tertiary Lymphoid Structures (TLS) Organized immune aggregates in tumors are often associated with favorable outcomes.
TILs (Tumor-infiltrating lymphocytes) Immune cells found in tumors, including cytotoxic T cells and Tregs.
VEGF (Vascular Endothelial Growth Factor) Potent angiogenic factor secreted by Tregs that promotes tumor vascularization.
